# Seasonal growth dynamics and yield potential of biomass sorghum in the Southeastern US

**DOI:** 10.1186/s12870-025-08032-1

**Published:** 2026-01-10

**Authors:** Tanumoy Bera, Yubin Yang, Lloyd T. Wilson, Fugen Dou, Joseph E. Knoll, Hamid Araji, William L. Rooney, Jesse I. Morrison, Brian S. Baldwin, John L. Jifon, Alan L. Wright, Dennis C. Odero, Hardev S. Sandhu

**Affiliations:** 1 Texas A&M AgriLife Research and Extension Center, 1509 Aggie Drive, Beaumont, TX 77713 USA; 2 Department of Ecosystem Science and Management, University of Wyoming, 1000 E. University Avenue, WY 82071 Laramie, USA; 3 Texas A&M University, College Station, TX 77843 USA; 4 Crop Genetics and Breeding Research Unit, USDA-ARS, 115 Coastal Way, Tifton, GA 31793 USA; 5 Department of Soil and Crop Sciences, Texas A&M University, College Station, TX 77843 USA; 6 Department of Plant and Soil Sciences, Mississippi State University, Mississippi State, MS 39762 USA; 7 Texas A&M AgriLife Research and Extension Center, 2415 E Hwy 83, Weslaco, TX 78596 USA; 8 Indian River Research and Education Center, University of Florida, 2199 South Rock Road, Fort Pierce, FL 34945 USA; 9 Everglades Research and Education Center, University of Florida, 3200 East Palm Beach Road, Belle Glade, FL 33430 USA

**Keywords:** Energy crop, Stalk density, Stalk height, Biomass growth curve, Yield penalties

## Abstract

**Background:**

The southeastern United States holds immense potential for producing cellulosic feedstocks to support the emerging biofuel industry. However, the development of a viable cellulosic biofuel sector depends on consistent, site-specific, and seasonally available biomass supply. Biomass sorghum has emerged as a promising annual feedstock, but understanding its growth dynamics and environmental sensitivities is essential for optimizing yield and supply logistics.

**Methods:**

A four-year, multi-location study was conducted across six sites in the southeastern US to assess the influence of genotype, environment, and management on biomass sorghum growth and productivity. The objectives were to: (1) quantify the growth and biomass dynamics of biomass sorghum under different environments in the Southeast US and 2) estimate early harvest yield penalties based on its seasonal biomass growth patterns.

**Results:**

Stalk density and plant height varied significantly across sites, years, and genotypes, reflecting strong genotype × environment interactions. Biomass accumulation followed a sigmoid growth pattern, with differences in heat unit requirements and the number of days to reach maximum biomass yield. Northern sites exhibited faster biomass accumulation but shorter growing seasons and higher early harvest penalties of up to 25%. End-of-season biomass ranged from 9.3 to 21.7 Mg ha⁻¹, with site accounting for the greatest source of variation, followed by site × year interaction.

**Conclusions:**

This study reveals strong spatiotemporal variability in biomass sorghum growth and yield across environments. The results emphasize the need for site-specific genotype selection, management strategies, and harvest scheduling to minimize yield losses and enhance feedstock reliability. These insights contribute to optimizing biomass sorghum production and strengthening sustainable bioenergy systems in the southeastern US.

**Graphical Abstract:**

Seasonal growth, early harvest penalties, and end-of-season yield of biomass sorghum in the southeastern US

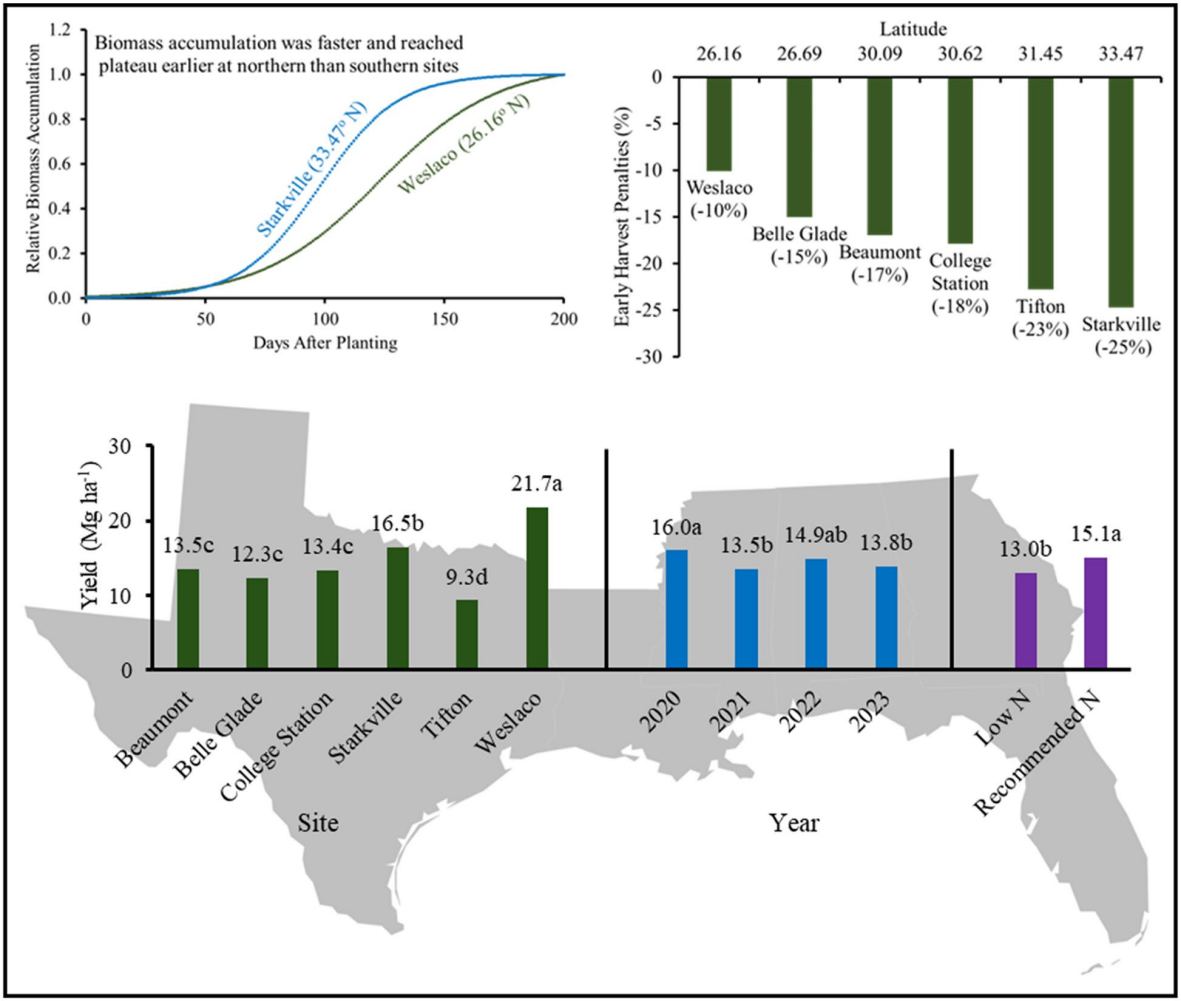

**Supplementary Information:**

The online version contains supplementary material available at 10.1186/s12870-025-08032-1.

## Introduction

The United States, Brazil, the European Union, and Argentina are the four largest biofuel producers in the world [1]. The 2023 Billion-Ton Report concluded that the United States has the potential to produce at least 1.2 billion Mg of biomass annually from agricultural, forestry, waste, and algal materials [2]. This biomass could displace approximately 265 billion liters or 30% of the country’s transportation liquid fuels consumption by 2050, without adversely affecting food production. Among the biomass resources, purpose-grown energy crops could produce 361 million Mg under the mature market medium scenario, while resulting in a 31% increase in total farm market net revenue. Among the purpose-grown energy crops, switchgrass (*Panicum virgatum* L.), energycane (*Saccharum* hybrid), and Miscanthus (*Miscanthus × giganteus*) are identified as the most promising perennial herbaceous crops, while biomass sorghum (*Sorghum bicolor* L. Moench), also known as energy sorghum or high fiber sorghum, has been identified as the most promising annual herbaceous crop [2].

Biomass sorghum hybrids are photoperiod-sensitive, offering a prolonged growing season and enhanced biomass potential, accumulating biomass into late September to early October, which corresponds to the autumn season [3, 4]. Previous studies have reported a wide range of biomass sorghum yields across the continental US, typically between 8 and 28 Mg ha⁻¹, depending on site, genotype, and management practices [5–8]. In Texas, biomass potential ranged from 5.8 to 16.6 Mg ha⁻¹ under dryland and irrigated conditions [6, 9], while in Florida, sweet sorghum produced 14.9–16.5 Mg ha⁻¹ with variable N inputs [10]. Multi-year studies in North Carolina and Illinois reported yield ranges of 8.1–27.7 Mg ha⁻¹ and 7.1–28.4 Mg ha⁻¹, respectively [11, 12]. A large multi-location study found maximum yields ranging from 24.6 Mg ha⁻¹ in Kansas to 41.1 Mg ha⁻¹ in North Carolina, emphasizing the influence of edaphic and climatic conditions on productivity [13]. Notably, Illinois produced 15.8% more biomass than Texas for the same genotypes [14]. Except for Texas, most of the studies on biomass sorghum yields have been conducted in the north-central US. Systematic studies on biomass sorghum in the Southeast US are lacking.

Furthermore, most biomass sorghum studies focus on end-of-season yields. However, for year-round operation of biorefineries, a continuous supply of feedstock is necessary. Thus, either harvesting must be spread over several months prior to and following normal harvest windows, or harvested biomass must be stored as off-season supply. Assessing biomass availability for early harvest requires seasonal biomass dynamics, which is lacking in the literature. Harvesting biomass sorghum prior to maturity is expected to result in early harvest yield penalties as reported by Yang et al. [15] for energycane. The objectives of this study were to 1) quantify the growth and biomass dynamics of biomass sorghum under different environments in the Southeast US and 2) estimate early harvest yield penalties based on its seasonal biomass growth patterns.

### Materials and methods

### Experiment sites

**Fig. 1:**
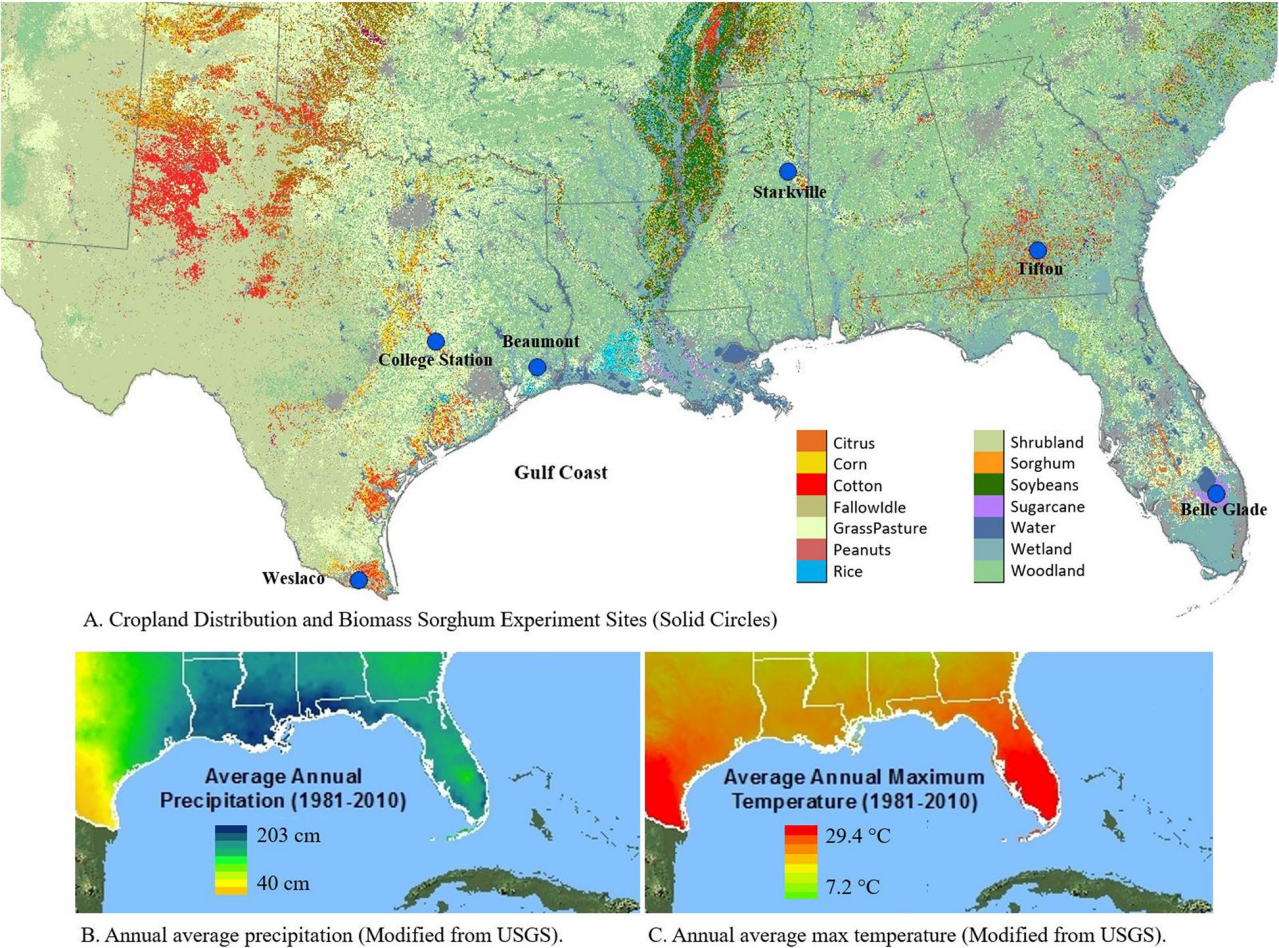
Field experiment sites (**A**) (Modified from Yang et al. 2011), average annual precipitation (**B**) (Modified from USGS 2016), and annual average max temperature (**C**) (Modified from USGS 2016) in the southeastern US

Field experiments were conducted at six locations in the southeastern US, including Beaumont, College Station, and Weslaco, Texas; Starkville, Mississippi; Tifton, Georgia; and Belle Glade, Florida (Fig. 1). These locations represent the range of environmental and production conditions in the southeastern US. Beaumont is characterized by high clay soil, high rainfall (~ 1600 mm/year), and mild winter with occasional short freezing in December or January. College Station is characterized by clay loam soil, approximately 1020 mm annual rainfall, and moderate winter. Weslaco has sandy clay loam soil and a subtropical climate with normally frost-free winter and low annual rainfall of 600 mm. Tifton is characterized by loamy sand soil with low organic matter, approximately 1200 mm rainfall, and winter with occasional freezing periods. Starkville has sandy loam soil, 1400 mm average annual precipitation, and winter with periodic freezing periods. Belle Glade has a histosol with more than 30% organic carbon (C), 1350 mm average annual rainfall, and winter with no freezing periods. Details of environmental conditions for each site are included in Table 1. The six study sites represent a range of humid subtropical to tropical climates across the southeastern U.S. Generally, these locations experience hot summers with average highs above 32 °C (90 °F) and mild to cool winters. Weslaco and Belle Glade are among the warmest sites, with minimal winter temperature drops, while Starkville and Tifton experience more seasonal variation. This range in temperature patterns provides a diverse environmental backdrop for evaluating biomass crop performance across latitudes.

**Table 1:** Summary of environmental conditions, field activities, and nitrogen rates at six experimental sites

Location	Year	Planting date	*N* rate (kg ha^− 1^)	Harvest date	Growing season solar radiation (MJ m^− 2^)^b^	Growing season degree days (^o^Cd)^c^	Growing season rainfall (cm) ^b^	Growing season potential evapotranspiration (cm) ^b^
Beaumont, TX	2020	24 Apr	112 & 280^a^	7 Oct	2199	3123	80.02	73.71
	2021	30 Apr	56 & 168 ^a^	22 Oct	2257	3187	103.06	71.87
	2022	2 Apr	56 & 168 ^a^	24 Sep	2318	3345	56.76	84.75
	2023	19 Apr	56 & 168 ^a^	2 Oct	2196	3346	50.58	86.74
College Station, TX	2020	21 May	112	13 Oct	1897	2909	28.49	83.23
	2021	15 May	112	18 Oct	2037	3080	51.46	90.97
	2022	10 May	112	28 Oct	2214	3577	24.55	105.04
	2023	14 Jun	112	9 Oct	1508	2798	2.13	75.20
Weslaco, TX	2020	9 Jun	61	30 Oct	1803	2905	22.93	81.81
	2021	28 May	61	15 Jan	2779	4137	66.95	77.70
	2022	4 Apr	61	16 Dec	3156	4798	47.40	125.68
	2023	24 Apr	61	12 Dec	2848	4577	35.0	120.64
Starkville, MS	2020	12 May	56 & 224 ^a^	9 Nov	2329	2860	71.54	93.78
	2021	27 May	56 & 224 ^a^	27 Oct	1989	2629	84.24	84.01
	2022	19 May	56 & 224 ^a^	28 Nov	1761	2446	42.08	75.36
Belle Glade, FL	2020	17 Jun	73	9 Nov	1803	2810	110.36	51.46
	2021	27 May	134	8 Oct	1723	2542	67.44	49.15
	2022	8 May	73	22 Nov	2471	3584	79.76	83.41
	2023	18 Apr	73	21 Nov	2709	3912	107.66	83.77
Tifton, GA	2020	26 May	280	5 Nov	2083	2848	41.96	83.16
	2021	4 May	280	10 Oct	2323	3005	63.48	93.64
	2022	18 Apr	51	4 Oct	2232	2990	33.08	93.77
	2023	12 Apr	280	9 Sep	1974	2557	53.76	73.75

^a^Two nitrogen rates were applied in Beaumont and Starkville

^b^Growing season is defined from planting to October 15 or harvest date whichever is earlier

^c^For a detailed description of the growing degree days methodology, please refer to the Materials and Methods section

### Field experiments

A randomized complete block design with four replicated blocks was used in all sites. Beaumont and Starkville had treatment combinations of 3 genotypes × 2 nitrogen (N) rates, while all other sites had treatment combinations of 3 genotypes × 1 N rate. Each site applied an optimal N rate based on local recommendation, while Beaumont and Starkville also included a low N rate treatment. Plot sizes varied across sites and years, ranging from 0.04 to 0.09 hectares. Plots in Weslaco were equipped with a drip irrigation system, ensuring biomass production under non-limiting water conditions. Other sites were either rainfed or received a single supplemental irrigation.

Treatments were arranged so that drainage from one plot did not flow through other plots. Row spacing was 0.76 m for all sites except for Tifton GA, which had a 0.91 m row spacing. Sorghum seed was treated with Concep, Apron XL and Maxim (safener, fungicide and insecticide, respectively) and drill-planted. Planting and harvesting information are summarized in Table 1.

End-of-season biomass yield was obtained by machine-harvesting each plot, with wet weight recorded with a weight wagon or scale. A random sample of approximately 1.5 kg of shredded biomass from each plot was collected, weighed and oven-dried at 65˚ C to constant weight for estimation of dry matter and moisture content.

### Seasonal crop growth and biomass dynamics through destructive sampling

For each location, two 6.1-meter sections of the middle two rows of each plot were marked with flags. The number of stalks in each section was counted approximately monthly from planting. Representative plants were randomly selected for destructive sampling to estimate plant height and dry mass of culms and leaves. Plant height was measured from the base of the stalk just above the soil surface to the uppermost leaf base with a visible collar. Plant organs were separated by type, oven dried for 7–14 days in a forced air oven at 65^o^ C and weighed.

### Biomass growth curve regression

Aboveground seasonally accumulated biomass was fit to logistic functions.

1 $$\:Biomass=Y\left(1+e\left(k*\left(CDD-b\right)\right)\right)^{-1}$$

2 $$\:Biomass=Y\left(1+e\left(k*\left(DAP-b\right)\right)\right)^{-1}$$

Cumulative growing degree days from planting is CDD (^o^CD), *Y* is maximum biomass at each site (Mg ha^− 1^), *b* is a scaling parameter that indicates the value of CDD where the biomass accumulation is 50% of the maximum, and *k* is a shape parameter that controls the slope of the curve at the inflection point. Larger values of *k* result in a steeper curve, while smaller values cause the curve to increase more gradually. Cumulative Degree Days are simply the sum of daily growing degree days (GDD) over a period of time, providing an integrated measure of heat units that a crop has received during its growing period. GDD is calculated daily using the formula: GDD = (Tmax + Tmin) / 2 – Tbase, where T_max_ is the daily maximum temperature, T_min_ is the daily minimum temperature, and T_base_ is the base or threshold temperature below which crop growth does not occur [17]. For sorghum, the base temperature is typically around 8 °C [18]. If the calculated average temperature falls below the base temperature, the GDD for that day is set to zero. Maximum temperature is capped at 35 °C to avoid overestimating growth under extreme heat. This method offers a standardized approach to track crop development across different environments. In Eq. 2, instead of CDD, days after planting (DAP) was used to regress biomass as well. Both regressions were conducted to determine whether using CDD or DAP provides a better fit.

### Statistical analysis

Analyses of variance (ANOVA) were conducted to assess the effects of site, year, genotype, N level, and their interactions on stalk density, stalk height, stem biomass proportion, and end-of-season yield using the SAS GLM procedure (SAS, 2015). When significant effects were detected, treatment means were differentiated using Tukey’s multiple comparison test at the significance level of α = 0.05 [19]. Regression analysis of biomass growth rates was performed using the logistic distribution function in [20]. Analysis of variance indicated significant effects of site and year on biomass yield. To determine the main environment covariates associated with site and year, we calculated two sets of environmental variables, including 1) soil and 2) weather factors during the crop season. Soil factors included soil texture (sand, clay, and silt %), soil organic matter, and soil pH. Weather factors included temperature, rainfall, solar radiation, and reference evapotranspiration. Stepwise regression in SAS GLMSELECT (SAS 2015) was used to identify the most significant variables impacting biomass sorghum yield [21].

### Bimass sorghum genotypes

Five biomass sorghum hybrids were grown in field experiments from 2020 to 2023, including TAM08010, TAM17501 and TAM17651 in 2020 and TAM08010, TAM08005 and TAM08001 in 2021–2023. Replacement of genotypes TAM17650 and TAM17501 in 2021 by TAM08001 and TAM08005 was based on breeder’s recommendation and overall feedstock characteristics. All genotypes were photoperiod sensitive hybrids but vary in stalk thickness and leaf to stem biomass ratio. Hybrids TAM17501 and TAM08010 have thinner stalks and greater leaf to stem biomass ratios while TAM08001 and TAM08005 have larger stems and fewer leaves which often shed as the season progresses, a desired characteristic as a biofeedstock. TAM17651 is intermediate to the others in phenotype. Seed was produced by the Texas A&M AgriLife Research Sorghum Breeding Program at locations near Bushland TX and processed by the Texas A&M AgriLife Research Foundation Seed Service. When seed are planted after the spring equinox, they will not initiate reproductive growth until just prior to fall equinox which allows up to 180 days of vegetative growth, depending on the planting date [16].

## Results

### Effect of site, year, genotype and N level on stalk density and height

Among the main effects, only site and genotype significantly influenced the average final stalk density, accounting for 22.5% and 3.4% of the variability, respectively (Table 2). Year and N level had no significant influence, contributing to less than 1% of the variability. However, all interaction effects were significant. The primary interaction was site × year, accounting for 40.5% of the variability, followed by site × genotype (12.8%), with a combined effect of 63.3%. All other interaction effects contributed to less than 15% (Table 2).

**Table 2:** Analysis of variance (ANOVA) on the effect of site, year, genotype, and nitrogen on stalk density, heights, and stem biomass proportions

Source	Density			Height			Stem biomass proportion		
	DF	Pr > F	Variance (%)	DF	Pr > F	Variance (%)	DF	Pr > F	Variance (%)
Site	5	< 0.0001	22.5	5	< 0.0010	27.5	5	< 0.0001	38.9
Year	3	0.1709	0.5	3	< 0.0001	10.2	3	< 0.0001	0.6
Genotype	4	< 0.0001	3.4	4	< 0.0001	2.2	4	0.0004	0.5
N Level	1	0.8727	0.0	1	< 0.0001	3.1	1	< 0.0001	0.4
Site×Year	13	< 0.0001	40.5	13	< 0.0001	42.2	13	< 0.0001	19.0
Site×Genotype	20	< 0.0001	12.8	20	0.0030	2.6	20	0.5644	0.4
Site×N Level	1	0.0014	1.0	1	< 0.0001	1.3	1	0.0427	0.1
Year×Genotype	4	< 0.0001	3.7	4	0.0100	0.9	4	0.1077	0.2
Year×N Level	3	0.0009	1.6	3	< 0.0001	6.8	3	< 0.0001	0.6
Genotype×N Level	4	0.0005	1.9	4	0.6350	0.2	4	0.2037	0.1
Site×Year×Genotype	16	0.0012	3.8	16	0.0340	1.5	16	0.0012	0.9
Site×Year×N Level	2	0.0226	0.7	1	0.8960	0.0	1	0.2103	0.0
Site×Genotyp×N Level	4	0.0018	1.6	2	0.2910	0.3	2	0.3864	0.0
Year×Genotyp×N Level	4	< 0.0001	5.1	4	0.0130	0.9	4	0.5276	0.1
Site×Year×Genotype×N Level	2	0.0463	0.6	2	0.0400	0.4	2	0.1602	0.1

**Fig. 2:**
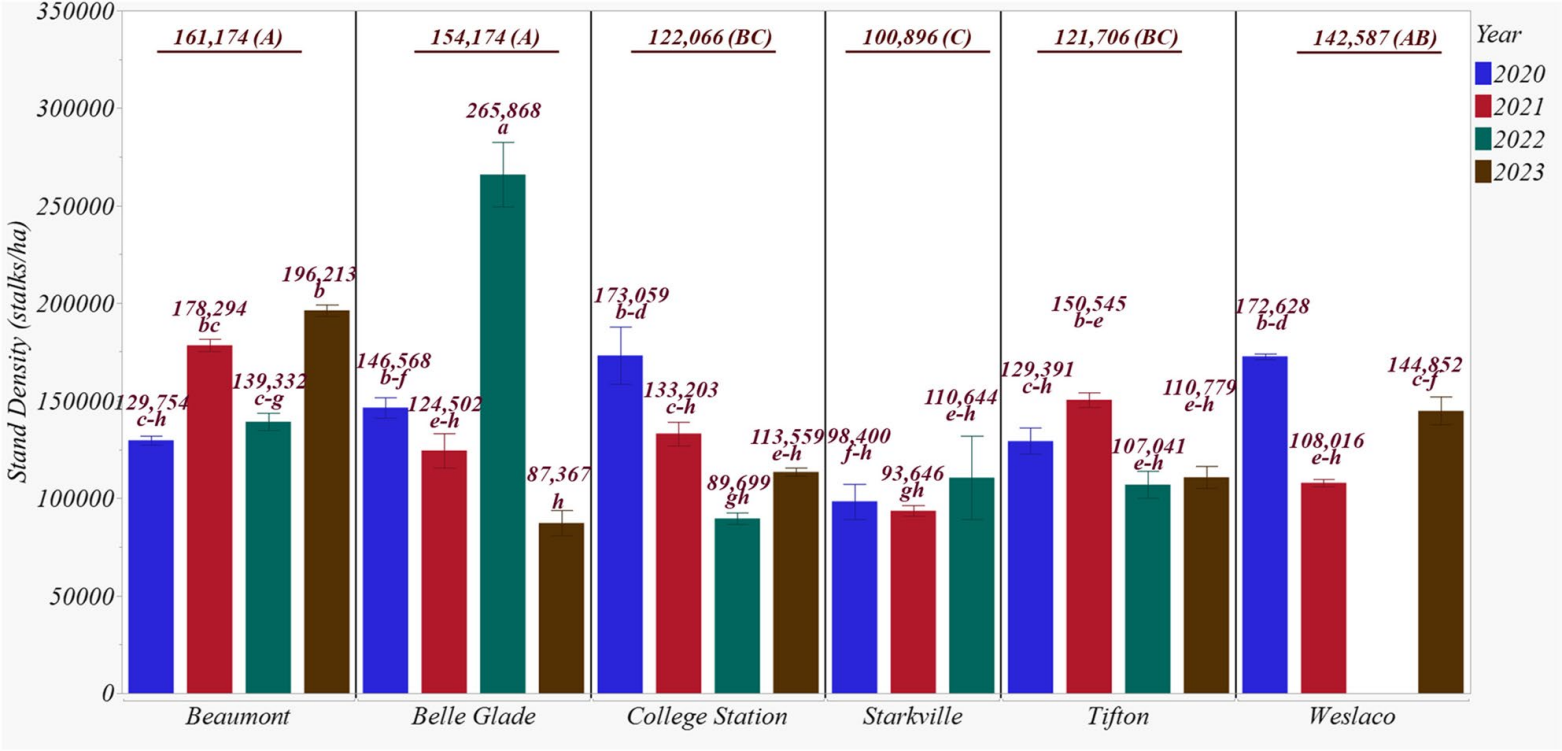
End of season biomass sorghum stalk density in 2020–2023 across six experimental sites. Stand densities having the same lowercase letter are not significantly different from each other at 0.05 with Tukey’s HSD multiple comparison test. Underlined values represent the average stalk density for each site, with different capital letters indicating significant differences among sites at *P* < 0.05, as determined by Tukey’s HSD multiple comparison test

Beaumont had the highest stalk density (161,174 stalks ha⁻¹), followed by Belle Glade (154,174 stalks ha⁻¹), though the difference was not statistically significant. Starkville recorded the lowest density at 100,896 stalks ha⁻¹, which was significantly lower than all other sites (Fig. 2). Weslaco, College Station, and Tifton exhibited intermediate densities of 142,587, 122,066, and 121,706 stalks ha⁻¹, respectively. Among genotypes, TAM08001 had the highest stalk density at 183,734 stalks ha⁻¹, while TAM17651 had the lowest at 129,025 stalks ha⁻¹.

The site × year interaction had the greatest influence on stalk density (Fig. 2). This was evident from the highest density recorded at Belle Glade in 2022 (265,868 stalks ha⁻¹), which contrasted sharply with one of the lowest densities at the same site in 2023 (87,367 stalks ha⁻¹). Beaumont showed significantly higher densities in 2021 and 2023 compared to 2020 and 2022. At College Station, the highest and lowest densities were observed in 2020 and 2022, respectively. Starkville did not show significant differences among years, while Tifton had its highest density in 2021. Weslaco recorded its greatest density in 2020, similar to College Station.

Plant height demonstrated a typical sigmoid pattern, with rapid mid-season growth before leveling off toward the end of the season. Table 2 summarizes the effects of site, year, genotype, N level, and their interactions on end-of-season stalk height. Among the main effects, site accounted for the most variability (27.5%), followed by year (10.2%), with both being significant. In contrast, genotype and N level explained only 2.2% and 3.2% of the variability, respectively. Similar to stalk density, interaction effects significantly influenced stalk height, except for genotype × N level and site × year × N level, which were not significant. The site × year interaction had the greatest influence, explaining 42.2% of the variability.

**Fig. 3:**
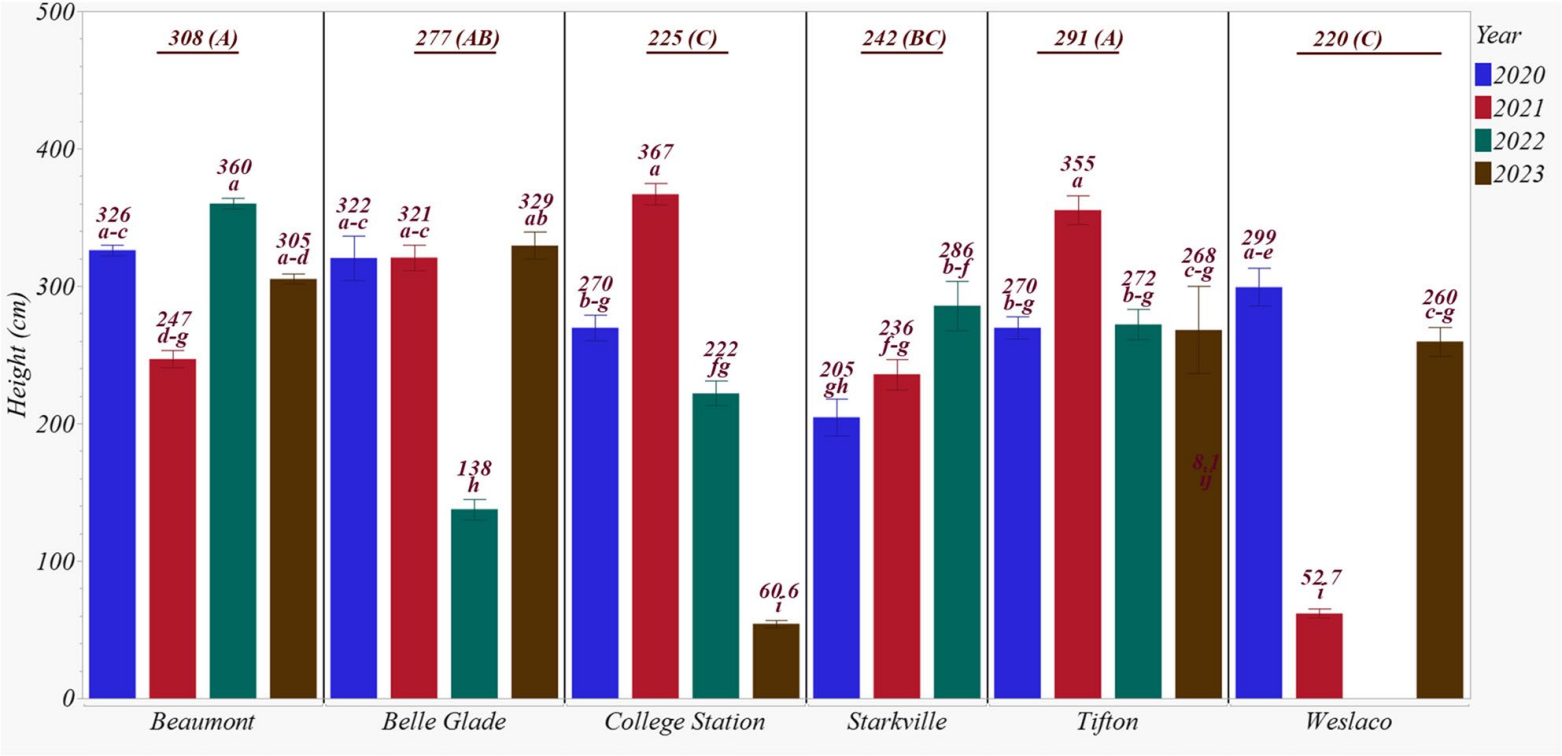
End of season biomass sorghum stalk height in 2020–2023 across experimental sites. Heights having the same lowercase letter are not significantly different from each other at 0.05 with Tukey’s HSD multiple comparison test. Underlined values represent the average stalk height for each site, with different capital letters indicating significant differences among sites at *P* < 0.05, as determined by Tukey’s HSD multiple comparison test

Beaumont recorded the tallest stalks, averaging 308 cm, followed by Tifton (291 cm) and Belle Glade (277 cm) (Fig. 3). These sites did not differ significantly from Beaumont. Weslaco had the shortest average stalk height at 220 cm, although this was not significantly different from College Sation (225 cm) or Starkville (242). The trends in stalk height across years varied considerably among sites, making the site × year interaction most significant (Fig. 3). A closer examination showed that Belle Glade in 2022, College Station in 2023, and Weslaco in 2021 had some of the shortest stalk heights recorded. For other site × year combinations, biomass sorghum generally reached heights of 250 cm or more, some exceeding 350 cm.

### Seasonal dynamics of biomass accumulation

**Fig. 4:**
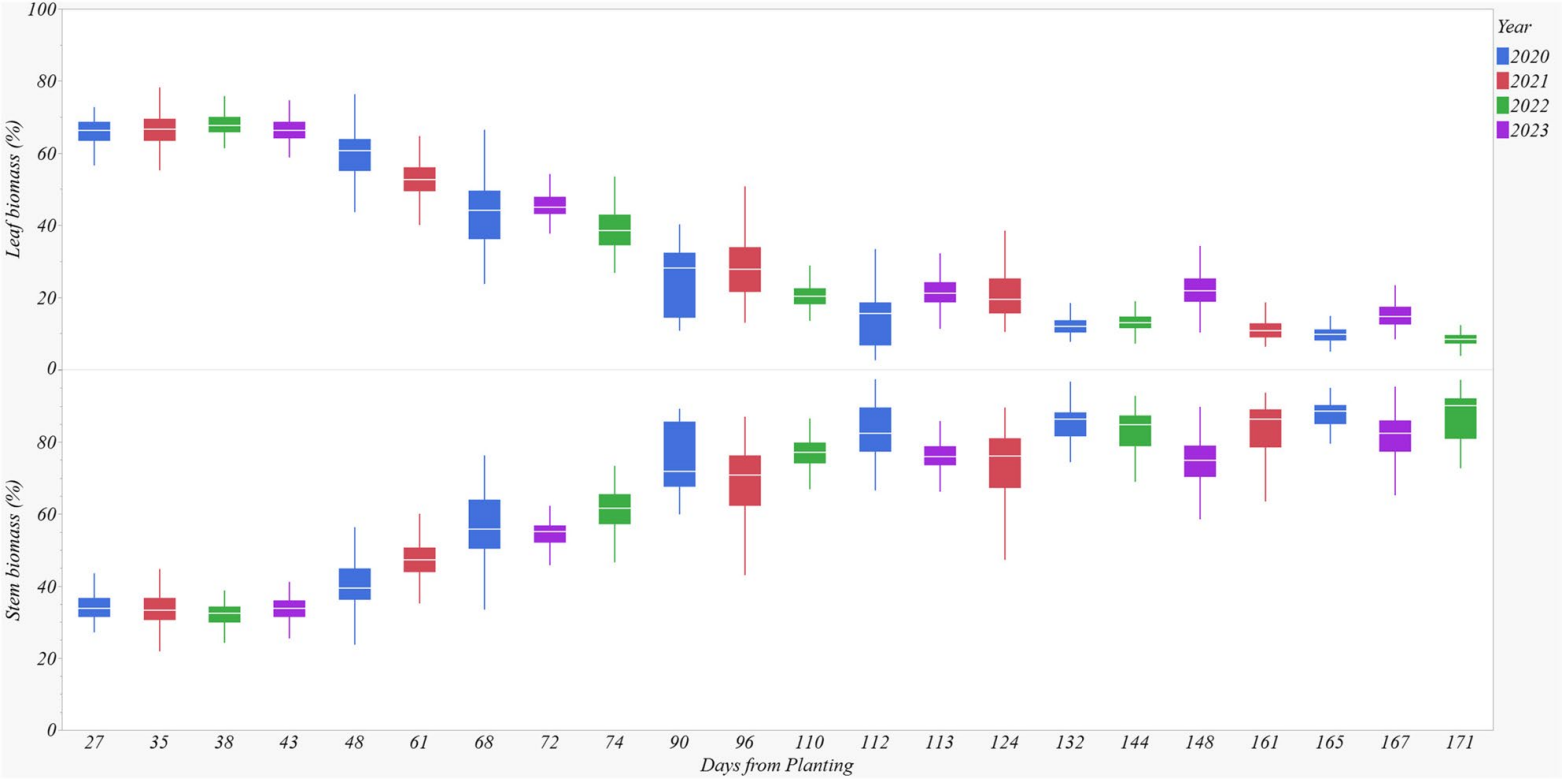
Seasonal dynamics of leaf and stem biomass fractions of biomass sorghum in Beaumont in 2020–2023 (Data pooled over three genotypes). Leaf and stem biomass fractions were expressed as a percentage (%) of the total above-ground biomass, which consisted of both leaf and stem biomass per stalk

There was a notable seasonal shift in above-ground biomass allocation to leaves and stems. Early in the season, leaves accounted for 60–80% of the biomass, but this proportion decreased to ~ 20% toward season’s end (Fig. 4). Conversely, the stem biomass percentage increased from ~ 20% to 60–80% by the end of the season (Fig. 4). This trend was consistent across all sites and years (Data is shown only for Beaumont). For the final destructive sampling, site significantly influenced the proportion of stalk biomass, explaining 38.9% of the variability (Table 2). Year, genotype and N level also had a significant effect, contributing to 0.6, 0.5 and 0.4% of the variability, respectively.

Among the interaction effects, the most prominent interaction was the site × year interaction, accounting for 19.0% of the variability (Table 2).

Belle Glade and Beaumont recorded the highest stalk biomass proportions in 2020, at 91% and 87%, respectively, which were significantly greater than those at other sites (Table 3). In 2021, Beaumont and Starkville achieved stalk biomass proportions of 87% and 84%, respectively. Beaumont maintained 87% stalk biomass in 2022, followed by Belle Glade (84%) and Starkville (81%). In 2023, Tifton recorded the highest stalk biomass proportion at 93% among all sites.

**Table 3:** Proportions of final stem biomass (%) at six experimental sites pooled over three genotypes for years 2020–2023

Sites	2020	2021	2022	2023
Beaumont, TX	87	87	87	81
Belle Glade, FL	91	67	84	83
College Station, TX	58	69	61	38
Starkville, MS	51	84	81	-
Tifton, GA	78	77	75	93
Weslaco, TX	74	77	-	69

### Seasonal biomass growth rate

**Fig. 5:**
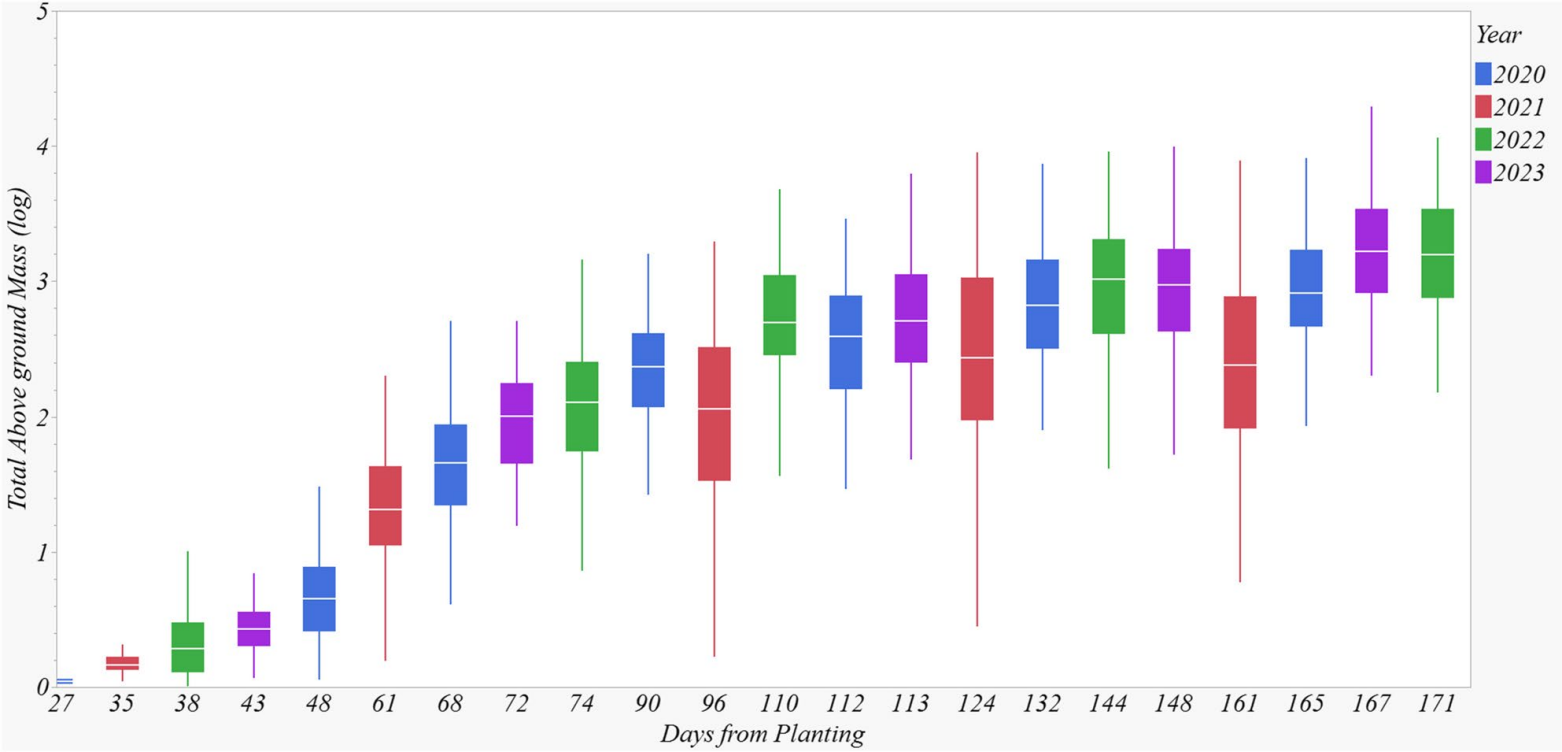
Seasonal aboveground biomass dynamics of biomass sorghum in Beaumont pooled over three genotypes. Aboveground biomass was calculated as average stalk biomass (leaf + stem) multiplied by stalk density on corresponding sampling date

**Fig. 6:**
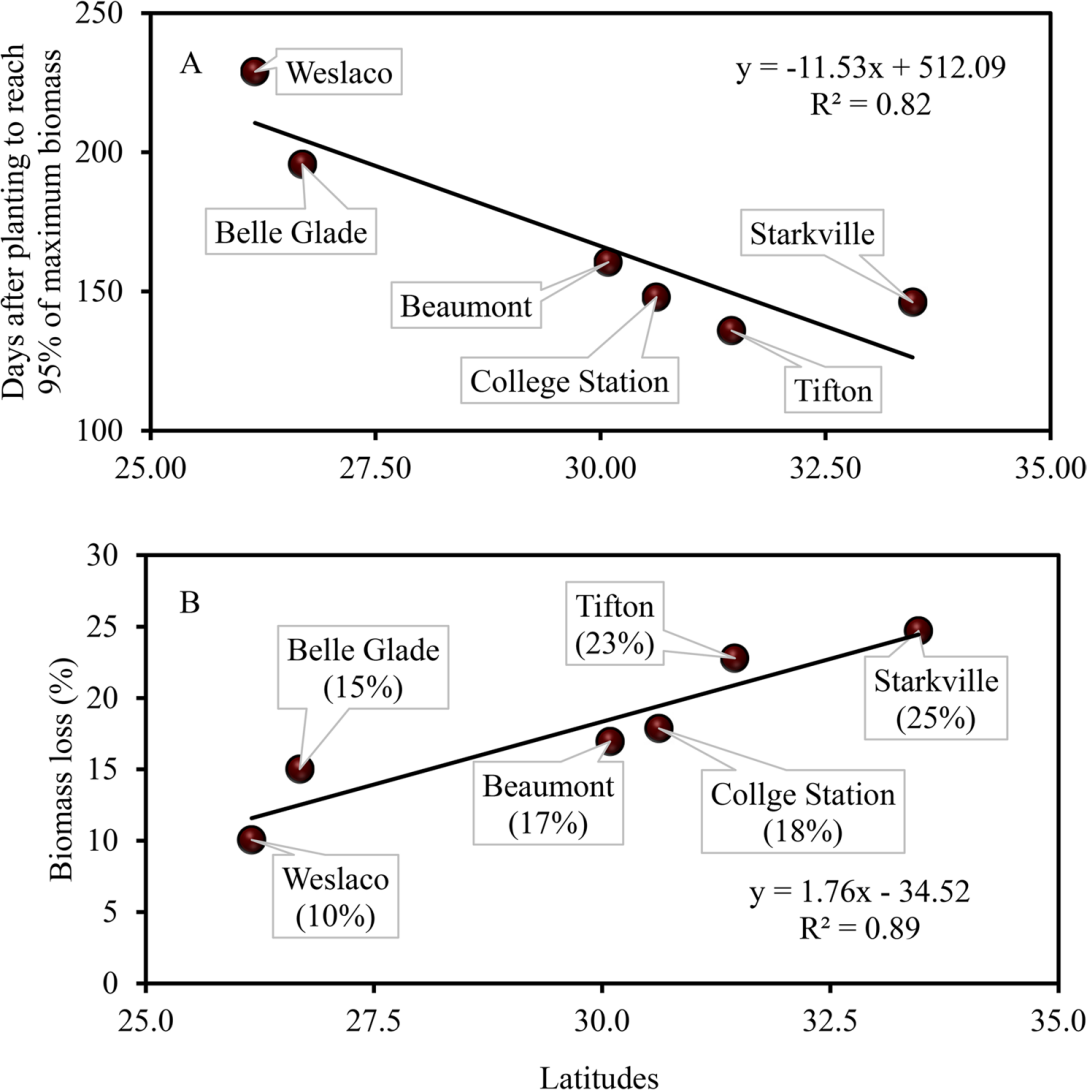
Effect of latitude on the number of days needed to reach 95% of maximum biomass from planting (**A**) and yield penalties associated with a one-month early harvest as a percentage of maximum biomass (**B**). Yield penalties and days to reach 95% of maximum biomass were calculated based on the biomass growth regression curves (Table 4)

The regression parameters for seasonal stalk biomass (Eq. (1)) are presented in Table 4. Stalk biomass exhibited a sigmoid growth pattern across all sites, with slow early growth, rapid increase during mid-season, and a gradual asymptotic slowdown towards the end of the season (Fig. 5). Southern sites, such as Belle Glade (123 days) and Weslaco (112 days), required a longer duration to reach 50% of maximum biomass accumulation compared to northern sites such as Starkville (98 days) and Tifton (85 days) (Table 4). Corresponding required heat units were 2, 281 and 2,334 ^o^CD for Belle Glade and Weslaco, and 1,805 and 1,515 ^o^CD for Starkville and Tifton (Table 4). The number of days required to reach 95% of maximum biomass accumulation were 229, 196, 161, 148, 146, and 136 for Weslaco, Belle Glade, Beaumont, College Station, Starkville, and Tifton, respectively (Fig. 6A). Notably, biomass sorghum at northern latitudes had a shorter growing season and a greater biomass accumulation rate (Table 4). A hypothetical harvest one-month prior to 95% maximum biomass would result in yield penalties of 10, 15, 17, 18, 23, and 25% for Weslaco, Belle Glade, Beaumont, College Station, Tifton, and Starkville, respectively (Fig. 6B). Therefore, early harvest yield penalty is more pronounced at northern sites compared to southern sites, indicating a narrower harvesting window for the northern sites and a wider window for the southern sites for a fixed percent early harvesting penalty. This underscores the critical importance of biomass supply logistics.

**Table 4:** Parameter estimates for biomass growth curves as functions of days after planting (DAP) or cumulative degree days since planting (CDD)

Site	Latitude(˚N)	Longitude(˚W)	Number of samples	Y Maximum biomass (Mg ha^-1^)	k(rate of plant growth)	50% biomass accumulation point	RMSE	*R* ^2^	Significance level
DAP									
Beaumont, TX	30.09	94.10	3309	19.81	0.0451	95 days	6.90	0.52	<0.0001
Belle Glade, FL	26.69	80.68	328	45.18	0.0318	123 days	19.64	0.41	<0.0001
College Station, TX	30.62	96.33	348	29.57	0.0473	86 days	8.77	0.60	<0.0001
Starkville, MS	33.47	88.81	569	18.04	0.0610	98 days	5.23	0.49	<0.0001
Tifton, GA	31.45	83.51	452	19.66	0.0578	85 days	7.36	0.60	<0.0001
Weslaco, TX	26.16	97.99	302	49.39	0.0320	112 days	9.71	0.51	<0.0001
CDD									
Beaumont, TX	30.09	94.10	3309	20.32	0.0021	1783 ^o^Cd	6.94	0.46	<0.0001
Belle Glade, FL	26.69	80.68	328	45.83	0.0016	2281^o^Cd	20.40	0.22	<0.0001
College Station, TX	30.62	96.33	348	29.36	0.0021	1742^o^Cd	9.08	0.42	<0.0001
Starkville, MS	33.47	88.81	569	18.46	0.0047	1805^o^Cd	5.39	0.49	<0.0001
Tifton, GA	31.45	83.51	452	19.64	0.0031	1515^o^Cd	7.38	0.49	<0.0001
Weslaco, TX	26.16	97.99	302	49.18	0.0007	2334^o^Cd	10.12	0.46	<0.0001

### Effect of site, year, genotype and N level on end-of-season biomass

End-of-season biomass was significantly influenced by site, year, genotype, and N level (Table 5), accounting for 31.1, 10.1, 6.1, and 7.6% of the observed variability, respectively. Among the interaction effects, site × year, site × genotype, and year × N level interactions were significant, explaining 29.0, 4.9, and 6.3% of the variability, respectively. Together, these four main effects and three two-way interactions explained a total of 67.1% of the observed variability. All other two-way, three-way, and four-way interaction effects were not significant.

**Table 5:** Analysis of variance (ANOVA) on the effect of site, year, genotype, and nitrogen (N) level on end-of-season biomass yield

Source	DF	Pr > F	Variance (%)
Site	5	< 0.0001	31.1
Year	3	< 0.0001	10.1
Genotype	4	< 0.0001	6.1
N Level	1	< 0.0001	7.6
Site×Year	15	< 0.0001	29.0
Site×Genotype	20	0.0138	4.9
Site×N Level	1	0.6080	0.0
Year×Genotype	4	0.5410	0.4
Year×N Level	3	< 0.0001	6.3
Genotype×N Level	4	0.2385	0.7
Site×Year×Genotype	20	0.8562	1.7
Site×Year×N Level	3	0.1719	0.7
Site×Genotyp×N Level	4	0.4576	0.5
Year×Genotyp×N Level	4	0.2994	0.6
Site×Year×Genotype×N Level	4	0.6712	0.3

**Fig. 7:**
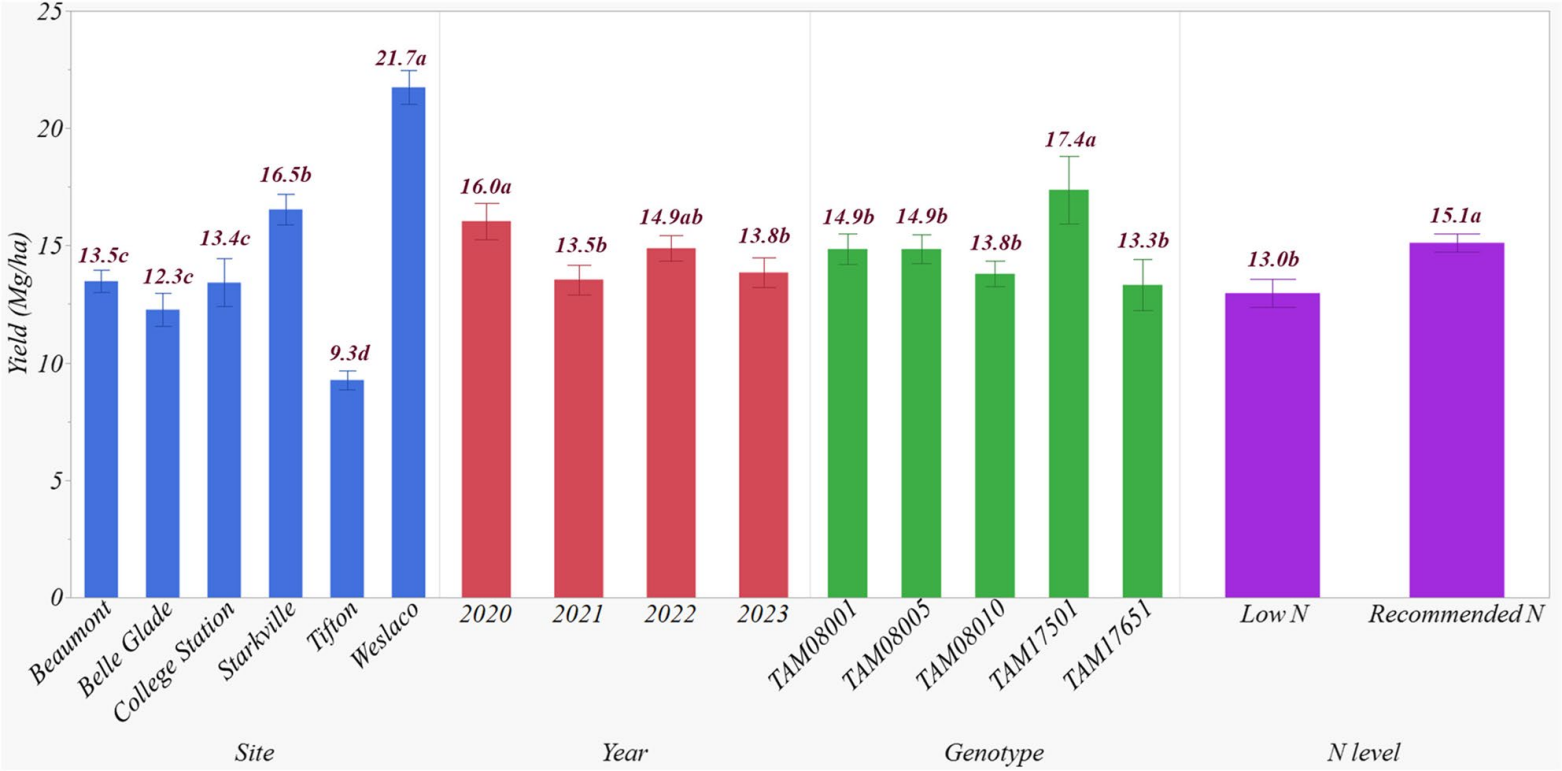
End-of-season aboveground biomass yield of biomass sorghum from 2020 to 2023 as influenced by the main effects of site, year, genotype, and nitrogen (N) level. Biomass yields having the same lowercase letter are not significantly different from each other at 0.05 with Tukey’s HSD multiple comparison test

Weslaco recorded the greatest biomass of 21.7 Mg ha^− 1^, followed by Starkville with 16.5 Mg ha^− 1^, which was significantly lower (Fig. 7). The lowest biomass was observed at Tifton (9.3 Mg ha^− 1^), which was significantly lower than Beaumont (13.5 Mg ha^− 1^), Belle Glade (12.3 Mg ha^− 1^), and College Station (13.4 Mg ha^− 1^). Biomass production was highest in 2020 (16.0 Mg ha^− 1^), followed by 2022, with no significant difference between 2022 and 2021 or 2023. However, biomass in 2021 and 2023 was significantly lower than in 2020. Among the genotypes, TAM17501 produced the highest average biomass of 17.4 Mg ha^− 1^ across all sites. Other genotypes in the study produced biomass that ranged from 13.3 to 14.9 Mg ha^− 1^. The recommended N application rate significantly increased biomass, producing 15.4% more biomass on average compared to the low N rate (Fig. 7) at Starkville and Beaumont.

**Fig. 8:**
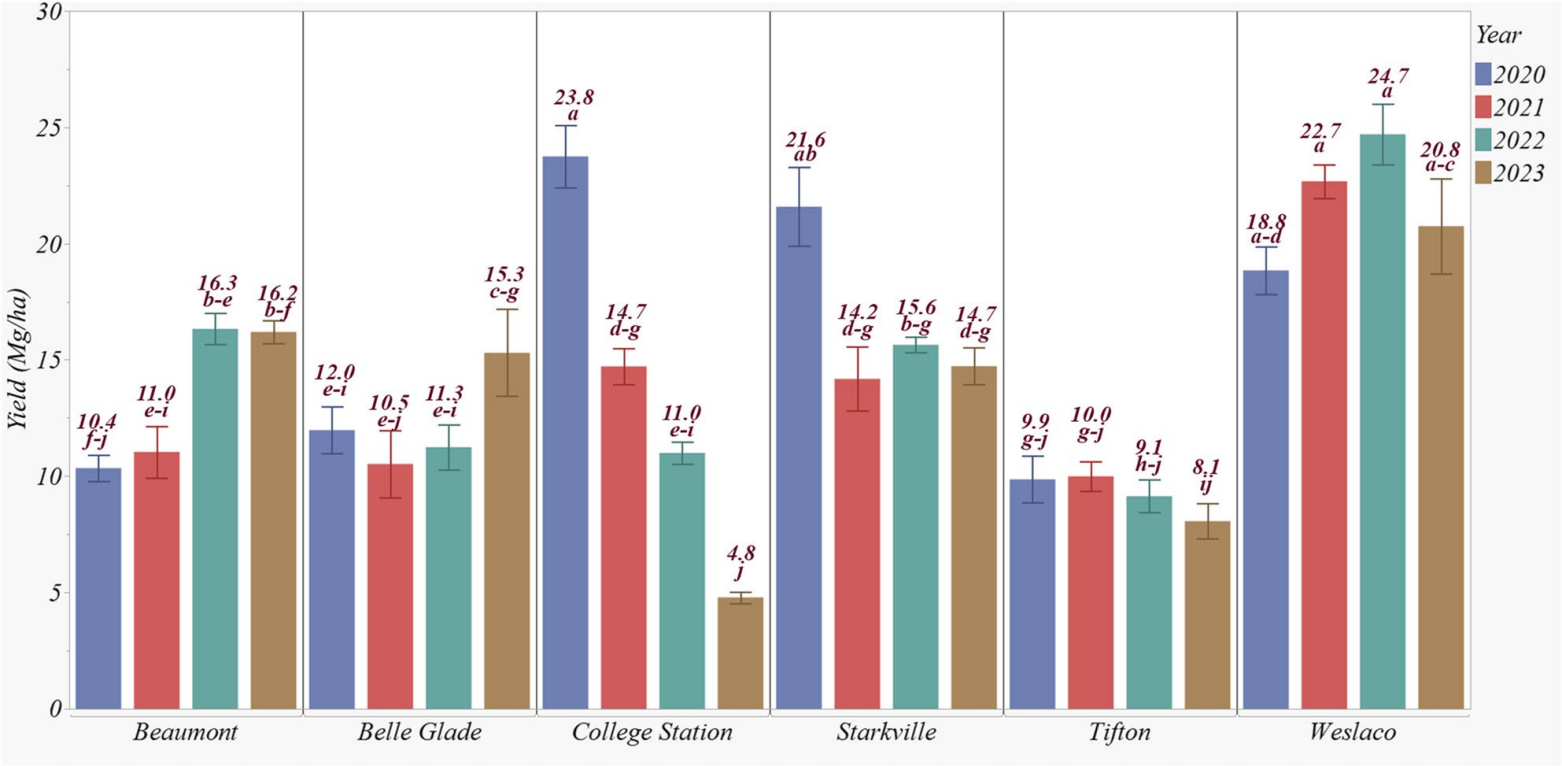
Effect of site × year interaction on end-of-season biomass yields for biomass sorghum from 2020 to 2023. Biomass yields having the same lowercase letter are not significantly different from each other at 0.05 with Tukey’s HSD multiple comparison test

Among the interaction effects, the site × year interaction accounted for the greatest explained variability, driven by the differences in biomass across years at each site. For instance, Weslaco achieved the highest biomass in 2022 (24.7 Mg ha^− 1^), although the biomass differences between years at this site were not significant (Fig. 8). Similarly, Tifton showed no significant biomass differences among years, though overall biomass was significantly lower than that of Weslaco. At Starkville, 2022 had the highest biomass of 21.6 Mg ha^− 1^, significantly greater than the other years. Beaumont had greater biomass in 2022 and 2023 (16.3 Mg ha^− 1^ and 16.3 Mg ha^− 1^, respectively), which were significantly greater than in 2020 and 2021. Belle Glade produced its greatest biomass in 2023 (15.3 Mg ha^− 1^), significantly greater than the other three years. College Station, however, displayed a significant decline in biomass over the years, with 2020 showing the greatest biomass of 23.8 Mg ha^− 1^, which dropped to 4.8 Mg ha^− 1^ by 2023 (Fig. 8).

### Environmental factors contributing to end-of-season biomass

**Fig. 9:**
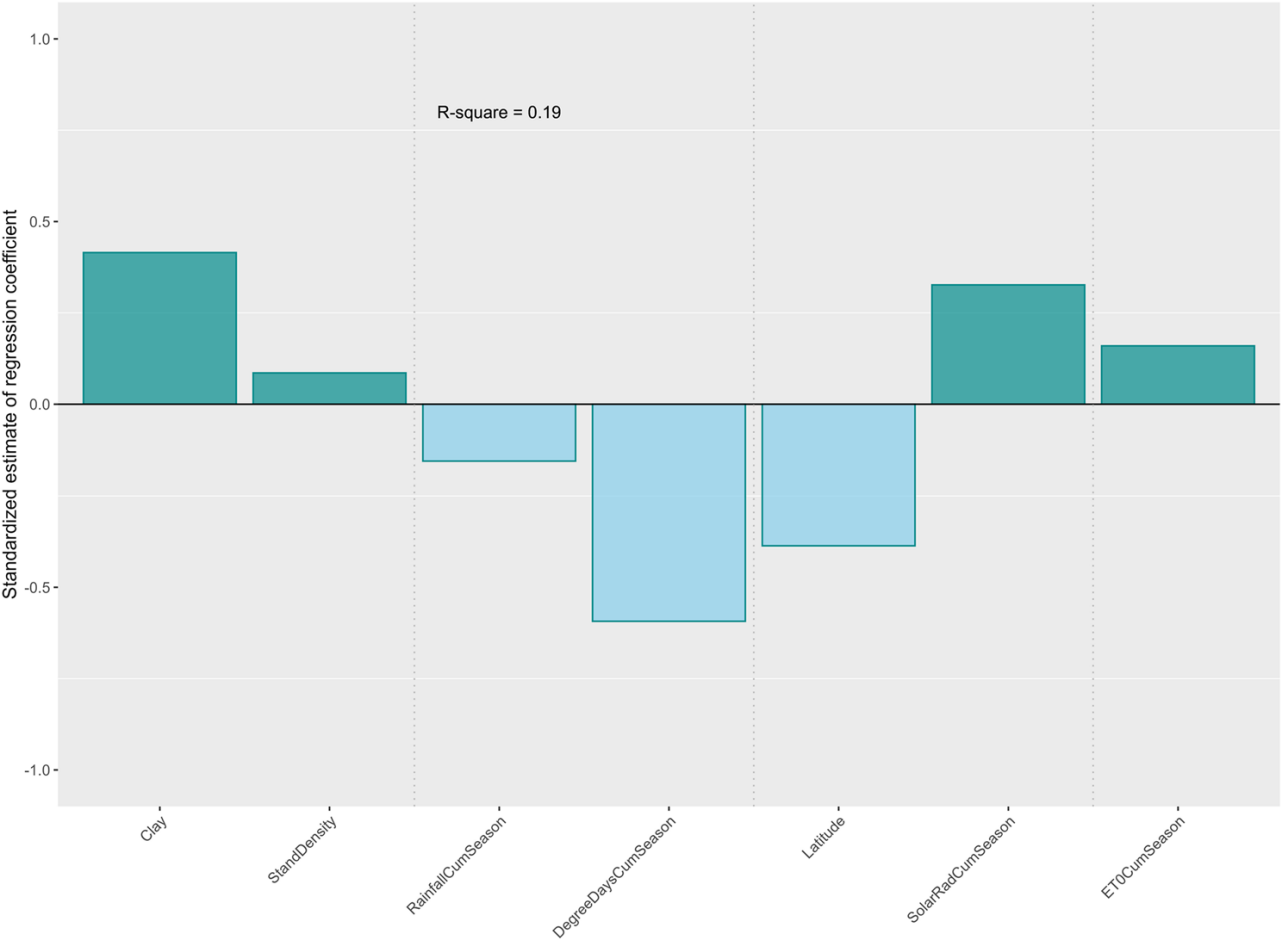
Impacts of environmental covariates on biomass sorghum yield (Bar plots showing significant environmental covariates through stepwise regression)

Figure 9 shows main environmental factors influencing biomass yields. Clay soil, stand density, growing season solar radiation and evapotranspiration had a positive effect on biomass sorghum yield, while growing season rainfall, thermal unit, and latitude had a negative effect. But these factors accounted for only 19% (${R^2}=0.19$ ) of the variance. A large majority of the variance is unaccounted for. Furthermore, interpretation of these impacts is not immediately clear.

## Discussion

### Factors impacting stalk density

The target stalk density for biomass sorghum experiments at all sites was 173,000 stalks ha⁻¹. But we observed considerable variability in stalk density across years and sites (Table 2), which is consistent with findings from other studies [22–27]. Highest stalk density was recorded in Belle Glade in 2022. This was likely due to the shallow water table within the biomass sorghum root zone, ensuring consistent moisture availability [28]. Similarly, Beaumont also exhibited high stalk density, suggesting that well-distributed moisture during planting helped maintain density. Planting season rainfall played a crucial role in stand establishment, as seen in Beaumont, where excessive rainfall resulted in low density in 2020 - over 25% of the total growing season precipitation fell within a month of planting. Such conditions likely exposed these genotypes to waterlogging stress, a phenomenon also reported by Heitman et al. [29]. Conversely, low stalk density in Starkville could not be solely attributed to water availability, as rainfall ranged between 42.08 and 84.24 cm over the four years. A likely cause could be lower seed planting rates. Biomass sorghum has high water use efficiency and drought tolerance, providing an advantage for cultivation on marginal lands under semi-arid conditions [4]. In a study by Steduto et al. [30], sorghum was the most water-use efficient species among several C4 grasses. Extensive research has delved into unraveling the mechanisms underlying sorghum’s drought resistance and the genetic effects governing it [31–33]. Genotypic differences likely contributed to the varying drought tolerance among sorghum hybrids, affecting stalk density under similar conditions.

### Factors impacting stalk height

In the present study, stalk heights exceeding 350 cm were indicative of greater biomass potential across many of the experimental sites over the years. In a previous study conducted across various locations in Texas, involving photoperiod-sensitive biomass hybrids, Hoffmann et al. [34] reported a mean stalk height of up to 312 cm, while Carvalho et al. [35] reported photoperiod-sensitive biomass sorghum stalk heights reaching greater than 400 cm. The authors emphasized that stalk height, along with stalk diameter, is a primary trait for predicting biomass across 20 sorghum genotypes. In our study, a significant but weak correlation between biomass and stalk height was observed. However, taller and thinner stalks were prone to lodging, posing challenges for biomass collection during machine harvest at the end of the season. Several authors have highlighted the issue of lodging in photoperiod-sensitive biomass sorghum hybrids, including Snider et al. [36] and Venuto & Kindiger [37] while others have suggested lodging resistance [34, 35] in such hybrids. In the present study, lodging with stalks exceeding a height of 300 cm was observed at several sites. The Southeastern US normally experiences high winds and rainfall during the hurricane season, which can exacerbate lodging. It is therefore imperative to factor in plant height and prioritize lodging-resistant biomass hybrids.

Genotypes TAM17650 and TAM17501 were replaced in 2021 by TAM08001 and TAM08005 based on breeder’s recommendation and overall feedstock characteristics. To assess whether genotype replacement introduces any bias in inter-year comparison, we conducted ANOVA analysis by excluding the two replaced genotypes and presented the results in Suppl. Table 1. Although the actual values of variance explained changed, the relative importance of each factor and their interactions remain similar to the analysis with all genotypes included, suggesting the validity of our analysis and conclusion.

### Seasonal biomass growth dynamics

Regression analysis for biomass growth can be conducted using days after planting or emergence [34, 38–41], or accumulated heat units [42]. Crop growth models such as DSSAT [18] and APSIM [43], simulate crop phenology and biomass growth based on thermal units [44], modulated by the crop’s sensitivity to photoperiod. It is postulated that a specific plant species will reach a particular phenological development stage when the required number of thermal units has been accumulated during the growing season, assuming all other growing conditions are identical [45]. Biomass sorghum may reach different phenological stages in different years, depending on seasonal temperatures. This suggests that relying solely on days after planting or emergence may not be suitable for consistently predicting plant development stages over the years, even under identical growing conditions. In this study, biomass sorghum was cultivated over a four-year period under diverse environmental and soil conditions (Table 1). The regression analysis conducted for a specific location, whether based on DAP or CDD, yielded similar results in terms of maximum potential yield. There was a direct linear relationship in RMSEs between CDD and DAP regressions, suggesting no discernible advantage of using CDD over DAP. Since field operations are usually based on calendar time, DAP was used for calculating early harvest penalties. Information on early harvest penalties is lacking in literature for biomass sorghum. Studies on energycane (*Saccharum* hybrid), a perennial bioenergy crop, have investigated both early harvest [15] and late harvest [46] penalties. If harvested on August 1 for energycane, Yang et al. [47] reported early harvest penalties of 60, 44, and 32% for Beaumont, Corpus Christi, and Weslaco, respectively. These penalties are significantly higher than those estimated in the present study, which range from 10% to 25%. This difference can be attributed to the fact that the early harvest penalties in the former study were 3 months prior to the maximum potential yield date.

### Factors impact final biomass yield

Environmental conditions, such as temperature, rainfall, and CDD, significantly influenced biomass sorghum growth and, ultimately, end-of-season biomass yield. This is evident from the wide range of biomass yields (8–25 Mg ha⁻¹) reported in the literature across the US [5, 6, 8, 29]. Among these environmental effects, temperature and CDD have been identified as major determinants of sorghum biomass production. Research suggests that for every 1 °C increase in temperature, forage sorghum yield can increase by approximately 0.53 Mg ha⁻¹ [48]. Given the similar genetic background and physiological characteristics of biomass sorghum, it is expected to perform better in warmer temperatures. This is supported by our findings, where the southernmost location, Weslaco, recorded the highest average biomass yield of 21.7 Mg ha⁻¹. Weslaco also experienced the highest CDD among all experimental sites, often exceeding 4000°CD days. Additionally, Weslaco was the only site where biomass sorghum was grown under non-limiting water availability due to the use of a drip irrigation system. Despite sorghum’s known drought tolerance, water availability remains a critical factor influencing productivity. Biomass sorghum hybrids are reported to be highly adaptable to warm conditions and can be planted once the soil temperature exceeds 8 °C [18]. However, while moderate temperature increases can enhance growth, excessive heat can negatively impact productivity. Sharma et al. [49] found that a 1 °C increase in maximum temperature above 25.9 °C can reduce yield by 7%. Similarly, Miller et al. [50] found a moderate 2 °C rise in growing season temperatures can lead to decreased biomass productivity. This highlights that the effect of temperature on biomass sorghum is not linear but is influenced by its magnitude and interactions with other environmental factors such as rainfall distribution.

Rainfall distribution primarily determines drought impacts on biomass sorghum production. For instance, in Beaumont in 2020, over 25% of the total seasonal rainfall occurred within one month of planting. This led to reduced plant density and lower biomass yield, despite higher total seasonal rainfall than in 2022 and 2023. Similar findings were reported by Heitman et al. [29], where excessive rainfall exceeding 30 cm within one month of planting in June created suboptimal conditions for crop establishment and negatively impacted end-of-season biomass. Biomass sorghum’s drought tolerance is attributed to its ability to maintain open stomata even at low water potential and across a wide range of leaf turgor pressures, contributing to greater biomass production [51]. The higher biomass yield at College Station compared to Belle Glade further supports the ability of biomass sorghum to perform well under suboptimal moisture conditions. College Station consistently received lower rainfall and higher PET than Bell Glade, yet it produced higher biomass yields. This suggests that biomass sorghum productivity is regulated not only by moisture availability but also by genetic diversity and adaptive physiological mechanisms.

Biomass sorghum has demonstrated high yield potential across various regions, with reported yields exceeding 25 Mg ha^− 1^ in several states, including Alabama [25], Illinois [52, 53], Iowa, Kansas, Kentucky, Mississippi [13], North Carolina [11, 13] and Texas [4, 13, 41, 42, 54, 55]. Modeling study had identified the southeastern US as particularly conducive to biomass sorghum production, presenting it as the greatest and most stable yielding region [7]. The present study encompassed five genotypes and six sites over four years, with biomass ranging between 9.3 and 21.7 Mg ha^− 1^. Comparisons with other studies, such as [56], suggest that differences in genotypes may contribute to variations in yield. Indeed, genotypic differences were evident in our study as well. Nitrogen fertilization plays a significant role in the biomass production of sorghum, with the optimal rate varying depending on several factors [8, 14, 29, 57, 58]. Research has shown that stems consistently make up the majority of the total crop biomass, typically around 70–85%, and N fertilization primarily influences the stem component [8, 57, 58]. In the present study, for Beaumont and Starkville, the recommended N rate led to a 15.4% increase in biomass yield. However, this increase must be evaluated considering both economic and environmental implications. Schetter et al. [14] recently concluded that N application rates beyond 112 kg ha⁻¹ do not result in higher biomass benefits. In contrast, the current study’s recommended N rates ranged from 168 to 280 kg N ha⁻¹, resulting in higher average yields. These findings highlight the need for a precise and calibrated N management strategy in bioenergy production, balancing yield potential, economic viability, and environmental sustainability.

In this study, N fertilizer gradients were applied only at Beaumont, Texas and Starkville, Mississippi, while fixed N rates were used elsewhere. This may have obscured complex interactions between N fertilizer and other factors, limiting the generalizability of conclusions. To access this limitation, we conducted ANOVA analysis using data only from the two sites and presented the results in Suppl. Table 2. Although the actual values of variance explained changed, especially for site, year, and site × year interactions, the relative importance of each factor and their interactions remain similar to the analysis with all data included, suggesting the validity of our analysis and conclusion.

Several factors need to be considered to achieve consistent high biomass sorghum yield. Biomass sorghum is very sensitive to excessive water in the seedling stage. Early bed preparation, sufficient bed height, and good water drainage are key to achieving uniform and good plant stand. Excessively high stand density will increase competition between plants and stunt their growth, resulting in lower biomass growth and yield, so proper seeding rate is key to achieve high yield. In addition, biomass sorghum is prone to lodging, especially under high N. Nitrogen should be applied at a moderate rate to promote biomass accumulation while avoiding excessive growth and potential lodging, but the present study’s unbalanced N treatments across locations limit our ability to define location specific optimal N rates. As such, numerical N recommendations cannot be directly derived from this data set. Beyond N management, water availability also plays a critical role in determining biomass sorghum performance. Proper irrigation can mitigate water stress, but late-season irrigation (after August) should be avoided since irrigation will exacerbate lodging due to excessive softening of the soil that holds the root crown.

Stepwise regression of main environmental covariates was only able to explain approximately 20% of the yield variability (Fig. 9). Inclusion of interactions only increased the explained variance to approximately 40% with the majority of the variance unexplained. Many of the environmental factors impact plant growth and development processes in a non-linear way over the crop season [18, 43]. The linear regression approach based on growing-season tally has limited capability in capturing the complex non-linear relationships in plant growth dynamics and final yield. In an attempt to better understand crop yield responses to diverse environments and provide better predictive capability, we have incorporated the results from this study to parameterize a biomass sorghum model based on the DSSAT-Sorghum Model [18], which can account for soil residual N, N losses, and plant demand in assessing optimal rates. This modeling framework is better suited for evaluating N response across diverse soil and climates and for determining regionally optimized N rates. This work is presented in a separate paper [59], which analyzes regional biomass sorghum yield responses and potential under different soil, weather, and management conditions across the Southeast United States.

### Implication for bioenergy development in the region

The updated Billion-Ton report [2] estimates substantial biomass production, projecting 361 million dry Mg from 31 million hectares of land, equating to a productivity of 11.7 Mg ha^− 1^. Comparatively, biomass yields in our study exceeded these projections averaging 15.6 Mg ha^− 1^ across sites and years. This highlights the potential of biomass sorghum cultivation across diverse environments and underscores its viability as a sustainable feedstock for industrial applications. Our regional simulation analysis [59] indicates that energycane is highly competitive along the coastal areas of the US Gulf Coast where there is abundant rainfall, while biomass sorghum is highly competitive in the inland areas of the Southeast US where there is less rainfall. These two energy crops can complement each other to support bioenergy development in the region and strengthen the region’s agriculture by promoting a bio-based economy.

Our multi-site and multi-year study highlighted substantial variation in biomass sorghum yields as impacted by local soil, weather, and production management. But this variation can’t capture the full range of yield variability due to changes in soil and weather conditions in the southeast US, especially impact of extreme weather events. A further study was conducted on regional analysis through computer simulations that can account for a wide range of soil and weather variability, using historic weather conditions [59]. This regional analysis can provide long-term risk assessment for bioenergy development in the region.

## Conclusion

In this four-year study on biomass sorghum production, we investigated the influence of environmental conditions and genotypes on stalk density, height, seasonal biomass dynamics, growth rate, and end-of-season yield. Our findings indicated significant variability in stalk density and height across experimental sites. Seasonal biomass dynamics underscored the importance of site-specific growth patterns. Biomass growth exhibited a sigmoid pattern, with variations in heat unit requirements and days to reach maximum accumulation. Notably, northern locations required fewer heat units and fewer days to reach maximum biomass, albeit with greater early harvest penalties of up to 25%. End-of-season yield varied significantly among sites and genotypes, ranging between 9.1 and 24.7 Mg ha^− 1^. Among the genotypes tested, Weslaco consistently produced the highest biomass, indicating its strong potential as a favorable site for biomass industry development in the region. Overall, our study provides insights into the complex interactions between site, genotype, and environment in optimizing biomass sorghum production for establishing sustainable bioenergy industries.

## Supplementary Information

Supplementary Material 1.

## Data Availability

Data supporting these findings are available within the article or upon request.

## Abbreviations

DAP: Days after planting
GDD: Growing degree days
CDD: Cumulative degree days
