# Supplementary material for: Seasonal growth dynamics and yield potential of biomass sorghum in the Southeastern US
Source: BMC Plant Biol. 2026 Jan 10;26:248. doi: 10.1186/s12870-025-08032-1 (PMC12882182; doi:10.1186/s12870-025-08032-1)
Supplement: Supplementary file 1 — Supplementary Material 1. [file 12870_2025_8032_MOESM1_ESM.docx]

**Supplementary Table S1**. Analysis of variance (ANOVA) on the effect of site, year, genotype, and nitrogen on stalk density, heights, and stem biomass proportions (2021-2023 data only)

|  | Density | | | Height | | | Stem biomass proportion | | |
| --- | --- | --- | --- | --- | --- | --- | --- | --- | --- |
| Source | DF | Pr > F | Variance (%) | DF | Pr > F | Variance (%) | DF | Pr > F | Variance (%) |
| Site | 5 | <.0001 | 16.5 | 5 | <.0001 | 12.2 | 5 | <.0001 | 39.1 |
| Year | 2 | 0.6062 | 0.2 | 2 | 0.0079 | 1.1 | 2 | 0.0189 | 0.3 |
| Genotype | 2 | 0.023 | 1.4 | 2 | 0.1702 | 0.4 | 2 | 0.0434 | 0.2 |
| N Level | 1 | 0.5149 | 0.1 | 1 | <.0001 | 2.2 | 1 | <.0001 | 0.9 |
| Site×Year | 8 | <.0001 | 27.0 | 8 | <.0001 | 36.8 | 8 | <.0001 | 22.3 |
| Site×Genotype | 10 | 0.0307 | 3.7 | 10 | 0.7347 | 0.8 | 10 | 0.4716 | 0.3 |
| Site×N Level | 1 | 0.2648 | 0.2 | 1 | 0.0005 | 1.4 | 1 | 0.0647 | 0.1 |
| Year×Genotype | 4 | 0.024 | 2.1 | 4 | 0.2296 | 0.6 | 4 | 0.1769 | 0.2 |
| Year×N Level | 2 | 0.4288 | 0.3 | 2 | <.0001 | 3.0 | 2 | 0.0011 | 0.5 |
| Genotype×N Level | 2 | 0.0915 | 0.9 | 2 | 0.6513 | 0.1 | 2 | 0.226 | 0.1 |
| Site×Year×Genotype | 16 | 0.8865 | 1.7 | 16 | 0.389 | 1.9 | 16 | 0.0096 | 1.1 |
| Site×Year×N Level | 1 | 0.1925 | 0.3 | 1 | 0.9056 | 0.0 | 1 | 0.2534 | 0.0 |
| Site×Genotyp×N Level | 2 | 0.1094 | 0.8 | 2 | 0.6933 | 0.1 | 2 | 0.4536 | 0.1 |
| Year×Genotyp×N Level | 4 | 0.1437 | 1.3 | 4 | 0.3202 | 0.5 | 4 | 0.6184 | 0.1 |
| Site×Year×Genotype×N Level | 2 | 0.421 | 0.3 | 2 | 0.2932 | 0.3 | 2 | 0.2181 | 0.1 |

**Table S2**. Analysis of variance (ANOVA) on the effect of site, year, genotype, and nitrogen (N) level on end-of-season biomass yield (Only data from Beaumont Texas and Starkville, Mississippi)

| Source | DF | Pr > F | Variance (%) |
| --- | --- | --- | --- |
| Site | 1 | 0.0002 | 5.6 |
| 6Year | 2 | <.0001 | 24.3 |
| Genotype | 2 | 0.3093 | 0.9 |
| N Level | 1 | <.0001 | 6.6 |
| Site×Year | 1 | 0.0017 | 4.1 |
| Site×Genotype | 2 | 0.3836 | 0.7 |
| Site×N Level | 1 | 0.0013 | 4.3 |
| Year×Genotype | 4 | 0.2615 | 2.1 |
| Year×N Level | 2 | <.0001 | 8.8 |
| Genotype×N Level | 2 | 0.6885 | 0.3 |
| Site×Year×Genotype | 2 | 0.2068 | 1.2 |
| Site×Year×N Level | 1 | 0.912 | 0.0 |
| Site×Genotyp×N Level | 2 | 0.7269 | 0.2 |
| Year×Genotyp×N Level | 4 | 0.3959 | 1.6 |
| Site×Year×Genotype×N Level | 2 | 0.345 | 0.8 |
